# Aberrant Cortical Layer Development of Brain Organoids Derived from Noonan Syndrome-iPSCs

**DOI:** 10.3390/ijms232213861

**Published:** 2022-11-10

**Authors:** Bumsoo Kim, Yongjun Koh, Hyunsu Do, Younghee Ju, Jong Bin Choi, Gahyang Cho, Han-Wook Yoo, Beom Hee Lee, Jinju Han, Jong-Eun Park, Yong-Mahn Han

**Affiliations:** 1 Department of Biological Sciences, Korea Advanced Institute of Science and Technology (KAIST), Daejeon 34141, Korea; 2 Graduate School of Medical Science and Engineering, Korea Advanced Institute of Science and Technology (KAIST), Daejeon 34141, Korea; 3 SoVarGen, Co., Ltd., Daejeon 34051, Korea; 4 Department of Pediatrics, Asan Medical Center Children’s Hospital, University of Ulsan College of Medicine, Seoul 05505, Korea

**Keywords:** Noonan syndrome, induced pluripotent stem cells, genetic modification, brain organoid, single-cell RNA sequencing, neurogenesis, cortical layer

## Abstract

Noonan syndrome (NS) is a genetic disorder mainly caused by gain-of-function mutations in Src homology region 2-containing protein tyrosine phosphatase 2 (SHP2). Although diverse neurological manifestations are commonly diagnosed in NS patients, the mechanisms as to how SHP2 mutations induce the neurodevelopmental defects associated with NS remain elusive. Here, we report that cortical organoids (NS-COs) derived from NS-induced pluripotent stem cells (iPSCs) exhibit developmental abnormalities, especially in excitatory neurons (ENs). Although NS-COs develop normally in their appearance, single-cell transcriptomic analysis revealed an increase in the EN population and overexpression of cortical layer markers in NS-COs. Surprisingly, the EN subpopulation co-expressing the upper layer marker SATB2 and the deep layer maker CTIP2 was enriched in NS-COs during cortical development. In parallel with the developmental disruptions, NS-COs also exhibited reduced synaptic connectivity. Collectively, our findings suggest that perturbed cortical layer identity and impeded neuronal connectivity contribute to the neurological manifestations of NS.

## 1. Introduction

Noonan syndrome (NS) is a genetic disorder diagnosed by multisystemic defects including characteristic facial dysmorphology, congenital heart disease, short stature, and developmental delay [1,2]. In addition to their typical facial and cardiac anomalies, many patients with NS commonly have neurological manifestations such as impairments in social cognition and language, learning difficulties, and mild intellectual disabilities [3,4,5,6]. To date, 13 different genes in the RAS/MAPK signaling pathway are associated with NS [7]. The majority of NS cases have various mutations in the *PTPN11* gene, which encodes the Src homology region 2-containing protein tyrosine phosphatase 2 (SHP2) [6].

Many studies have attempted to identify the etiology of NS-associated neurological symptoms in various SHP2 mutant mouse models. SHP2 D61G/+ mice have increased neuronal populations in the neonatal forebrain and hippocampus regions caused by perturbations in cell fate decisions [8]. Moreover, a variety of SHP2 mutant (SHP2 D61G/+, SHP2 D61Y/+, and SHP2 N308D/+) mice show synaptic abnormalities in hippocampal neurons, such as impaired synaptic plasticity and dysregulated glutamate receptor expression, leading to defective spatial learning and memory [9,10,11,12,13]. Therefore, these mouse models of NS have provided important insights into the neurological defects associated with this disorder. Nonetheless, since cortical development is different in its early stage between humans and rodents, mouse models cannot fully recapitulate neurodevelopmental disorders at higher levels of complexity [14]. Due to this limitation, findings in mouse models often cannot be translated to humans and drug targets discovered from these models predominantly fail to demonstrate similar efficacy in clinical trials [15]. As an alternative, brain organoids derived from human induced pluripotent stem cells (iPSCs) are used to study various neurodevelopmental diseases [16,17]. Brain organoids have uncovered the pathological mechanisms underlying neurodevelopmental disorders, including impaired neural progenitor proliferation in microcephaly, reduced neuronal migration in lissencephaly, the imbalance between excitatory and inhibitory neuronal populations in autism spectrum disorder, and altered cortical layer identity in schizophrenia [18,19,20,21]. These disease-specific brain organoids also provide a valuable drug screening platform for the translational medicine of diverse neurodevelopmental and neurodegenerative disorders [22,23]. Recently, NS patient-derived iPSCs (NS-iPSCs) were found to exhibit precocious gliogenesis during early cortical organoid development [24]. However, whether the SHP2 mutation causes aberrant neurogenesis during human cortical development has not yet been reported.

Here, we revealed neurodevelopmental defects in cortical layer formation by analyzing the single-cell transcriptome of NS-cortical organoids (COs). Although NS-COs form normal ventricular/subventricular zone-like structures during development, similar to WT-COs, single-cell transcriptomic profiles showed an increase in the glutamatergic neuronal population in NS-COs. A subset of the glutamatergic neuronal subpopulation in NS-COs co-expressed the deep layer marker CTIP2 and upper layer marker SATB2, indicating dysregulated cortical layer identity. Consistent with the layer phenotype, NS-COs showed reduced synaptic connectivity. The co-expression of CTIP2 and SATB2 and decreased neuronal connectivity in the NS-COs were restored when the SHP2 mutation in NS-iPSCs was corrected by gene editing. Collectively, our findings indicate that the SHP2 mutation impairs cortical layer development and neuronal connectivity in NS-COs during cortical development in vitro.

## 2. Results

### 2.1. Normal Development of NS-iPSCs into Cortical Organoids

**Figure 1:**
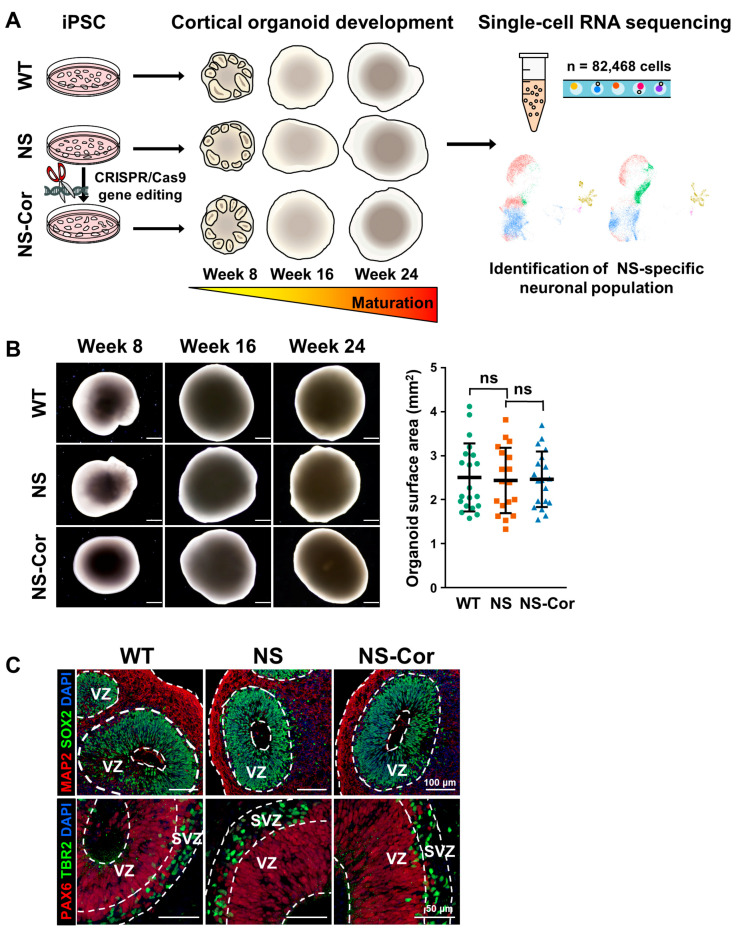
Normal development of NS-iPSCs to COs. (**A**) Schema of the experimental procedures. (**B**) Normal morphological development of NS-COs. No differences were observed in the surface areas of week 8-COs among the WT-, NS-, and NS-Cor-groups. The surface areas of 18–20 organoids in each group were measured using ImageJ and repeated independently three times. Data are presented as means ± SD. *p*-values were determined using the unpaired Student’s *t*-test. ns, not significant. Scale bars, 500 μm. (**C**) Inner structures of week 8-COs. Like WT- and NS-Cor-COs, NS-COs had an undisrupted precursor-rich structure. VZ, ventricular zone; SVZ, subventricular zone. Scale bars, 100 μm (upper panel); 50 μm (lower panel).

We previously reported that NS-iPSCs show abnormal gliogenesis during early neural development in vitro [24]. To understand whether the SHP2 mutation influences neurogenesis during the process of cortical development, transcriptomic signatures were serially profiled over time in developing human COs. To achieve this, COs were obtained at weeks 8, 16, and 24 during the differentiation of WT-iPSCs, NS-iPSCs, and the gene-corrected isogenic control cell line of NS-iPSCs (NS-Cor-iPSCs), and then subjected to single-cell RNA sequencing (scRNA-seq) analysis (Figure 1A). The NS patient donor had a heterozygous PTPN11 c.922 A > G genetic mutation and was diagnosed with both typical NS phenotypes (facial dysmorphism, short neck, short stature, and pulmonary stenosis) and neurological manifestations (borderline intellectual functioning, learning disability, and attention deficit hyperactivity disorder). To minimize the genetic background effects, the SHP2 mutation of NS-iPSCs was corrected using the CRISPR/Cas9 system (Figure S1A). The established NS-Cor-iPSCs contained the PTPN11 c.922 A nucleotide in both alleles (Figure S1B) and did not have any undesirable off-target mutations (Figure S1C) or karyotypic abnormalities (Figure S1D). All of the three iPSC lines used in this study (WT-, NS- and NS-Cor-iPSCs) expressed pluripotency-associated markers (NANOG, SOX2, Tra-1-60, Tra-1-81, and ALP) (Figure S1E). These iPSC lines normally developed into COs appearance-wise and had similar organoid sizes (Figure 1B and Figure S2A). Ventricular zone (VZ)- and subventricular zone (SVZ)-like structures were observed in developing week 8-COs regardless of their cell origin (Figure 1C). The expression of PTPN11 gene was not affected by the genetic mutation in both mRNA and protein levels during CO development (Figure S2B,C). However, SHP2 mutation increased the catalytic activity of the protein and consequently hyperactivated the MAPK pathway in NS-COs. Therefore, NS-iPSCs appeared to be as developmentally competent as WT- and NS-Cor-iPSCs even with the MAPK pathway hyperactivation, especially during their early development into neural precursors. Nonetheless, since some of the other encephalopathy cerebral organoid models have shown defective neuronal development even without significant abnormalities in their neural progenitor population [25], we hypothesized that the SHP2 mutation may influence the neuronal population during the developmental process of NS-COs. To test this hypothesis, we performed time-series scRNA-seq in WT-, NS-, and NS-Cor-COs.

### 2.2. Aberrant Glutamatergic Neurogenesis in NS-COs

**Figure 2:**
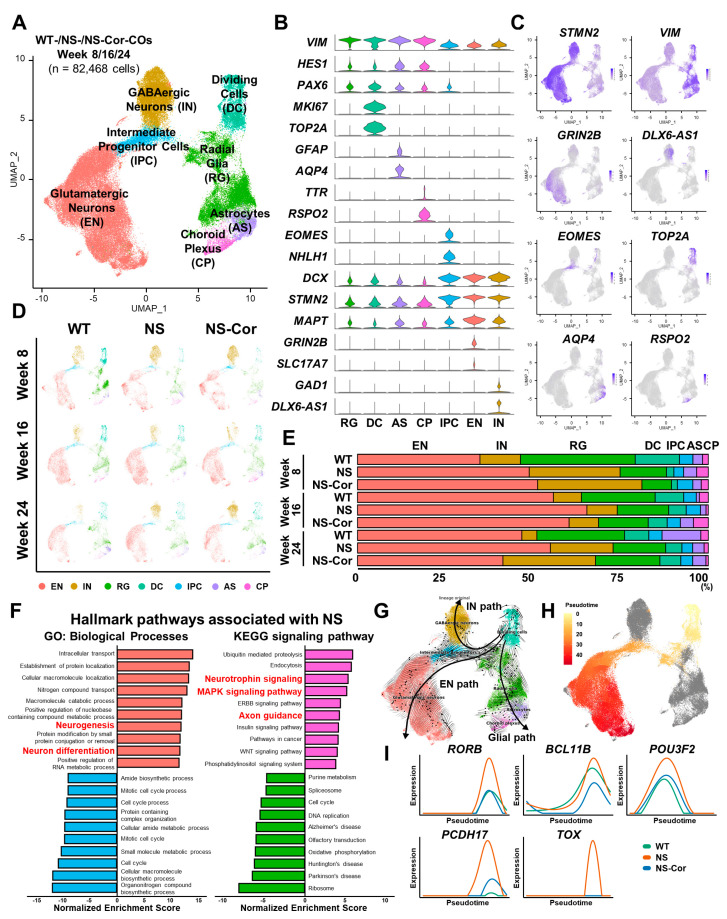
Single-cell transcriptomic analyses of COs. (**A**) UMAP plot of a total of 82,468 cells obtained under nine distinct experimental conditions (WT-/NS-/NS-Cor-COs, Week 8/16/24). Clusters were annotated into seven different cell types based on the expression of the cell type-specific genes. (**B**) Transcriptional expression of representative lineage- and cell type-specific genes in each cluster. (**C**) Distribution of cells expressing representative lineage- and cell type-specific genes in UMAP space. (**D**) Cell type distribution of COs according to the experimental conditions. (**E**) Proportions of cell types in the respective CO-groups. (**F**) Gene Ontology analysis of the top 10 hallmark pathways significantly enriched or reduced in NS-COs. (**G**) RNA velocity of COs. Three developmental pathways are depicted on the UMAP. (**H**) Pseudotime trajectory of cells lining the EN pathway. (**I**) Transcriptional expression of diverse cortical layer marker genes along the pseudotime.

After filtering out low-quality cells, a total of 82,468 cells were clustered in an unsupervised manner, and further visualized using uniform manifold approximation and projection (UMAP). Seven major clusters exhibiting similar differentially expressed genes (DEG) patterns were annotated by their respective cell types. The classified cell types were glutamatergic neuron (excitatory neuron, EN), GABAergic neuron (inhibitory neuron, IN), radial glia (RG), dividing cell (DC), intermediate progenitor cell (IPC), astrocyte (AS), and choroid plexus-like cell (CP) (Figure 2A). Neural precursor-associated genes (VIM, HES1, PAX6) were highly transcribed in RG, DC, AS, and CP, whereas neuronal lineage-associated genes (DCX, STMN2, MAPT) were transcriptionally enriched in the IPC, EN, and IN cell types (Figure 2B). Other cell type-specific markers were also specifically expressed in each cluster; MKI67 and TOP2A in DC, GFAP and AQP4 in AS, TTR and RSPO2 in CP, EOMES (TBR1) and NHLH1 in IPC, GRIN2B (NMDAR2B) and SLC17A7 (vGLUT1) in EN, and GAD1 and DLX6-AS1 in IN (Figure 2B). The expression profiles of additional marker genes in each cluster are shown in Figure S3A. When these transcriptional profiles were distributed in UMAP space, spatially restricted patterns of cell type-specific markers were found to be similar to those of the respective clusters (Figure 2C). These results corroborated the validity of our cell type annotation in the analysis of scRNA-seq data obtained from human COs. When the cell type composition was assessed at different developmental stages, the UMAP plot of respective CO samples exhibited similar distribution patterns (Figure 2D). Nonetheless, minor variations were detected in the cell type signatures between NS/NS-Cor- and WT-COs. These variations were attributed to differences in the genetic backgrounds of the sources of the COs and not to the patient-specific SHP2 mutation, highlighting the effectiveness of our genetic correction.

To determine whether the SHP2 mutation affected neuronal cell types, cell type proportions were assessed in WT-, NS-, and NS-Cor-COs at each time point. In all CO samples, ENs were the most abundant cell type (Figure 2E). Moreover, the proportion of ENs in NS-COs was higher than that in WT- or NS-Cor-COs across most cortical developmental stages (Figure S3B). Gene set enrichment analysis (GSEA) also revealed increased neurogenesis, neuronal differentiation, axon guidance, neurotrophin signaling, and MAPK pathway signaling in NS-COs (Figure 2F). Thus, it is conceivable that the increased EN population in NS-COs is associated with the SHP2 mutation. Next, we performed trajectory analysis to determine whether the transcription of EN-associated genes is disturbed in NS-COs. RNA velocity analysis revealed three distinct developmental pathways (EN, IN, and glial) in COs (Figure 2G), which resembled the neurogenic and gliogenic processes of the RG in the developing human cerebral cortex [26,27]. To analyze single-cell trajectory during glutamatergic neurogenesis, only cells lining the EN path were ordered in pseudotime using the Monocle 3 package (Figure 2H). Interestingly, various genes including RORB, BCL11B (CTIP2), POU3F2 (BRN2), PCDH17, and TOX, which are known to be expressed in a cortical layer-specific manner [28], were overexpressed in NS-COs throughout in vitro EN development (Figure 2I). However, the expression patterns of proliferation-, neural precursor lineage-, and neuronal lineage-associated genes in NS-COs were similar to those in WT-/NS-Cor-COs along the pseudotime (Figure S3C). We therefore concluded that the SHP mutation promotes increased EN differentiation and dysregulated cortical layer identity in NS-iPSCs during cortical organoid development.

### 2.3. Defective Cortical Layer Formation in NS-COs

**Figure 3:**
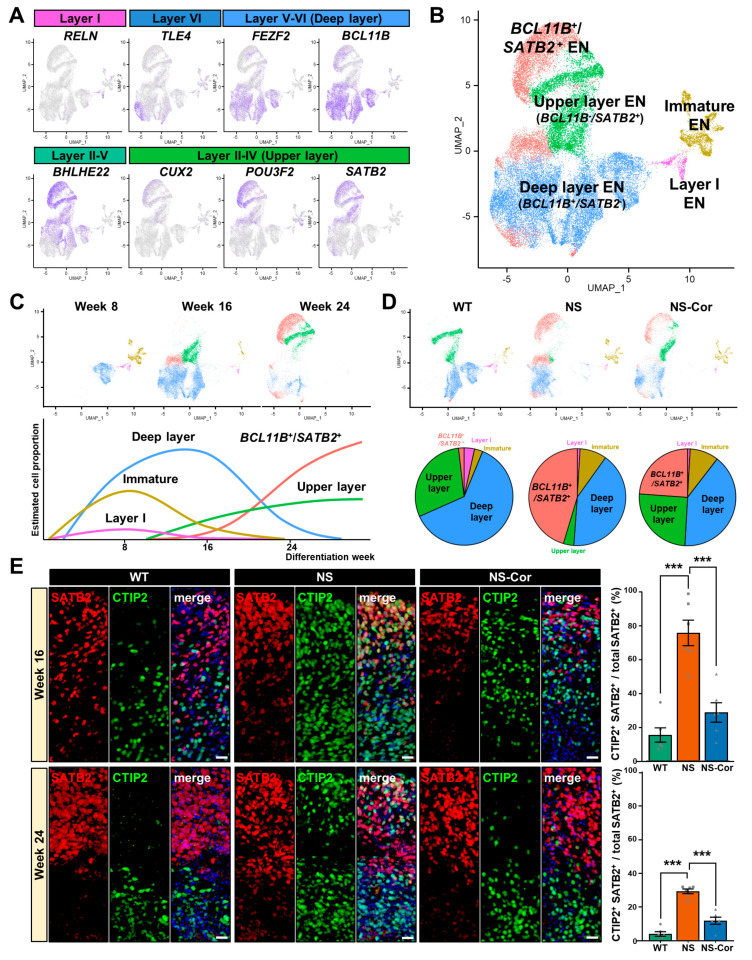
Disturbed regulation of cortical layer identity in NS-COs. (**A**) Distribution of the expression of cortical layer-specific genes in the EN subset. (**B**) UMAP plot of ENs. Clusters were annotated based on their respective layer-specific gene expression. (**C**) Laminar identity transition across cortical development. Schematic dynamics are drawn in accordance with the cell proportion at each time point. (**D**) Cortical layer distribution of respective COs. (**E**) Increased CTIP2^+^/SATB2^+^ population in week 16, 24-NS-COs. Numbers of CTIP2^+^/SATB2^+^ cells were counted in six organoids in each group with ImageJ. COs were obtained from three independent experiments. Data are presented as means ± SEM. *p*-values were determined with an unpaired Student’s *t*-test. *** *p* < 0.001. Scale bars, 20 μm.

To further investigate the aberrant development of ENs in NS-COs, we analyzed the expression profiles of various cortical layer marker genes in the EN subset. The distribution of the expression of the respective layer markers, including layer I marker (RELN), layer VI marker (TLE4), deep layer V-VI markers (FEZF2 and BCL11B), layer II- V maker (BHLHE22), and upper layer II-IV markers (SATB2, POU3F2, and CUX2), was grouped in UMAP space (Figure 3A). Surprisingly, we found that a large proportion of the EN population that expressed upper layer marker genes such as CUX2, POU3F2, and SATB2, co-expressed the deep layer marker gene BCL11B. Hence, the EN population was sub-classified into the following five groups: layer I EN, deep layer EN, upper layer EN, BCL11B^+^/SATB2^+^ EN, and immature EN (Figure 3B). As the COs developed, the population of both layer I EN and immature EN gradually decreased whereas the deep layer EN population increased until week 16. Thereafter, the upper layer EN and BCL11B^+^/SATB2^+^ EN populations increased until week 24 (Figure 3C, upper). The temporal dynamics of these EN identities are depicted in the schematic illustration (Figure 3C, lower). To determine whether the aberrant BCL11B^+^/SATB2^+^ EN population was associated with the SHP2 mutation, we analyzed the laminar compositions of ENs in the different COs. Intriguingly, the proportion of BCL11B^+^/SATB2^+^ EN population was highly increased whereas that of the BCL11B^−^/SATB2^+^ upper layer EN population was lower in NS-COs than in WT-COs (Figure 3D). Although the population of BCL11B^+^/SATB2^+^ ENs was higher in NS-Cor-COs than in WT-COs, their proportion was consistently lower than in NS-COs. Principal component analysis (PCA) of pseudo-bulked layers revealed a higher correlation between two SATB2-positive subtypes than between other EN populations (Figure S4A). These results suggest that BCL11B^+^/SATB2^+^ ENs may be produced instead of upper layer ENs during the cortical development of NS-COs. Among the DEGs between the BCL11B^+^/SATB2^+^ ENs and upper layer ENs, LMO4 expression, previously reported to interfere with the SATB2-mediated epigenetic repression of CTIP2 [29], increased in BCL11B^+^/SATB2^+^ ENs (Figure S4B). Furthermore, the downregulation of NR2F1, a transcription factor that determines the cortical caudal-rostral axis, and upregulation of CPNE8, a prefrontal cortex-specific upper layer marker [30,31], indicates that the signaling pathway changes caused by the SHP2 mutation override the molecular mechanisms of cortical arealization, resulting in NS-specific dysregulation of cortical layer identity. Immunostaining analysis confirmed that CTIP2^+^/SATB2^+^ co-positive cells were substantially higher in week 16 and 24 NS-COs than in WT/NS-Cor-COs (Figure 3E). In agreement with the scRNA-seq data, SATB2 protein was not expressed at the early developmental stage (week 8) in all samples (Figure S4C). Thus, we detected developmental abnormalities in NS-COs in which the excitatory neuronal population co-expressed CTIP2 and SATB2. Our findings demonstrate that the SHP2 mutation causes aberrations in cortical layer development during the development of NS-COs in vitro.

### 2.4. Impaired Neuronal Connectivity in NS-COs

**Figure 4:**
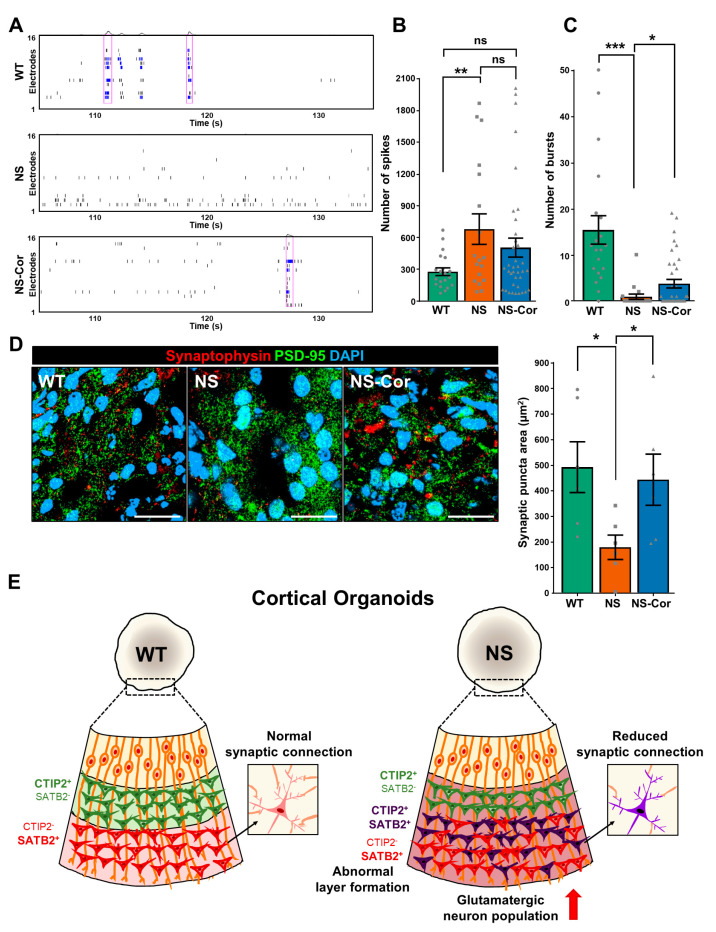
Aberrant neuronal connectivity in NS-COs. (**A**) Raster plot of extracellular spontaneous firing in week 24-COs. Blue lines indicate bursts and purple boxes represent network bursts. (**B**) Number of spikes in COs. Numbers of spikes and bursts were counted in 18–37 active electrodes. Active electrodes are defined as electrodes that exhibit more than 5 spikes per min. The number of spikes was analyzed using Axis navigator software. (**C**) Number of bursts in respective COs. The number of bursts was also analyzed using Axis navigator software. (**D**) Reduced area of synaptic puncta in NS-COs. Areas showing co-localization between synaptophysin and PSD-95 were counted in six organoids per group using Fiji software. COs were obtained from three independent experiments. Scale bars, 20 μm. (**E**) A schematic model of the neurodevelopmental defects in NS-COs. In (**B**–**D**), data are presented as means ± SEM. *p*-values were determined using an unpaired Student’s *t*-test. * *p* < 0.05; ** *p* < 0.01; *** *p* < 0.001.

Next, we conducted multi-electrode array (MEA) analysis to investigate the electrophysiological properties of NS-COs. Since neuronal electrical activity and network formation increase as the brain organoid develops [32,33], week 24-COs were subjected to MEA analysis. Spontaneous spikes were detected in all COs during MEA recording (Figure 4A). Despite higher firing frequencies (see the raster plot, Figure 4A), NS-COs rarely displayed bursts (synchronous action potential over a brief time period, blue bars) or network bursts (simultaneous bursts across multiple electrodes, purple boxes) (Figure 4A). The number of spikes per active electrode increased (Figure 4B), whereas the number of bursts was significantly lower in NS-COs than in WT-/NS-Cor-COs (Figure 4C). Network bursts were not detected in all NS-COs (Figure S5). Thus, it is conceivable that the SHP2 mutation causes electrophysiological disturbance in cortical neurons, specifically excessive firing and perturbed network formation. To further investigate the synaptic connectivity of NS-COs, the expression of the presynaptic marker synaptophysin and the postsynaptic marker PSD-95 was analyzed in week 24-COs. The area of synaptic puncta, identified by the co-localization of synaptophysin and PSD-95, was also significantly decreased in NS-COs (Figure 4D), consistent with the reduced burst frequency observed in MEA analysis. Together, our findings suggest that the SHP2 mutation impedes the formation of normal synaptic connections between excitatory neurons during in vitro cortical development.

## 3. Discussion

In this study, we demonstrated that aberrant cortical layer formation and impaired neuronal connectivity in glutamatergic neurons are associated with the neurological symptoms of NS. Single-cell transcriptomic analysis revealed that the EN population undergoes excessive neurogenesis, and that cortical layer formation is dysregulated during the development of NS-COs derived from NS-iPSCs. Furthermore, NS-COs were electrophysiologically dysfunctional due to reduced formation of synaptic connections. Thus, we conclude that the SHP2 gain-of-function mutation causes defective cortical neuron development in NS-COs.

Most patients with NS have below average IQs and a higher prevalence of seizure disorders and autism spectrum disorders [34]. To investigate these neurological defects in NS, various studies using NS-mouse models have been conducted. SHP2 D61G/+ mice show impaired hippocampal long-term potentiation caused by either increased surface expression of the AMPA receptor or NMDA receptor dysfunction [9,11]. EMX1-specific SHP2 D61Y/+ mice exhibit disrupted surface expression of a diversity of glutamate receptors in hippocampal neurons [10]. Although those studies were able to partially recapitulate the memory and learning disabilities in NS patients, the mechanisms underlying cognitive impairment and intellectual disability remain unclear. Since the cerebral cortex plays a primary role in cognition and intelligence, structural malformations or neuronal miswiring during cortical development are associated with epilepsy and intellectual disability symptoms in a variety of neurodevelopmental disorders [35,36]. Recently, the combined techniques of brain organoid culture and single-cell transcriptomic analysis have facilitated the identification of a diversity of neurodevelopmental abnormalities in neuropsychiatric disorders, such as the emergence of atypical neuron populations, immature neurogenesis, and disrupted neurodevelopmental trajectories in vitro [37,38,39,40]. Here, we also identified novel neurodevelopmental abnormalities in NS-COs using scRNA-seq analysis. NS-COs displayed a consistently higher proportion of ENs than WT-/NS-Cor-COs during development (Figure 2E and Figure S2B). Moreover, gene sets involved in neurogenesis and the MAPK signaling pathway were significantly enriched in NS-COs (Figure 2F). In mouse cortical precursors, transfection with SHP2 D61G upregulates the MAPK pathway and promotes neurogenesis [8]. Thus, our results suggest that activated MAPK signaling also contributes to the increase in the EN population in NS-COs. The MAPK signaling pathway also plays a crucial role in the normal development of cortical layers, especially CTIP2^+^ neurons in layer V [41]. Transcriptional profiles of several cortical layer markers were aberrant in NS-COs throughout EN development (Figure 2I). In parallel, an atypical EN population co-expressing the deep layer marker CTIP2 and upper layer marker SATB2 were specifically increased in NS-COs whereas upper layer EN population decreased (Figure 3). Collectively, our results indicate that the neurological manifestations of NS are associated with an expanded population of excitatory neurons and disrupted cortical layer identity during cortical organoid development. 

During embryonic development, six distinct cortical layers (layers I–VI) are formed sequentially in an inside–out fashion [42]. Aside from their laminar positions, neurons in each layer exhibit unique molecular signatures of various transcription factors, which further determine the distinctive characteristics of the respective layers [43]. Layer-specific transcription factors form a complex genetic network to specify the laminar identity of individual cortical neurons [44]. Since SATB2 epigenetically represses the expression of CTIP2, neurons co-expressing both genes are mainly found in the developing mouse cerebral cortex around E16.5, and their population gradually decreases by the early postnatal period [45,46,47,48]. Interestingly, the CTIP2^+^/SATB2^+^ EN population was consistently higher in NS-COs than in WT-COs, regardless of the developmental stage (Figure 3). Recently, cortical organoids with gene *DISC1* mutations were reported to exhibit aberrant co-immunostaining of upper layer makers (SATB2 and RORB) and deep layer markers (TBR1 and CTIP2) during cortical organoid development [21]. Since disorganized cortical layer formation is frequently observed in other neurodevelopmental disorders such as autism spectrum disorder [49,50], it is evident that the dysregulated co-expression of layer-specific markers in brain organoid models takes part in the abnormal neurological functioning in the patients. Therefore, our findings suggest the possibility that perturbations in the laminar identity of cortical neurons contributes to the neurological symptoms of NS patients.

CTIP2 and SATB2 directly regulate the axonal projections of cortical neurons in the developing cerebral cortex [51]. Deep layer corticofugal projection neurons fail to extend their axons into the corticospinal tract in CTIP2 knockout (KO) mice [52], while upper layer callosal projection neurons change their corpus callosum projection to corticospinal projection in SATB2 KO mice [45,46]. Furthermore, a small number of CTIP2^+^/SATB2^+^ co-positive neurons in the postnatal mouse brain show alterations in axonal guidance and electrophysiological properties [29]. Thus, it is possible that defective CTIP2 and SATB2 regulation contributes to impaired axonal projections of cortical neurons in NS-COs. In fact, NS-COs exhibited fewer neuronal connections and synapse formation than the control groups (Figure 4). Neuronal connectivity in the human brain is established by the coordinated regulation of axonal guidance and synapse formation [53]. Moreover, it has been proposed that disrupted cortical connectivity is associated with cognitive and behavioral symptoms in patients with autism spectrum disorder [54,55,56]. A recent fMRI study showed that NS patients have alterations in functional connectivity across diverse brain regions [57]. Collectively, our findings show that altered neuronal connectivity caused by the SHP2 mutation leads to impaired cognitive functions associated with NS.

Here, we propose a model to explain the defective neurodevelopment of NS-COs (Figure 4E). Using a single-cell transcriptomic approach, we found that neurogenesis and the determination of laminar identity were both impaired in NS-iPSCs during cortical organoid development. Aberrant co-expression of CTIP2 and SATB2 was predominantly observed in NS-COs, and was partially reduced in NS-Cor-COs. Moreover, the electrophysiological properties in NS-COs were altered, particularly in terms of disrupted synaptic connectivity. Although we did not perform studies on other NS-iPSC models with different mutations, our findings in SHP2 N308D mutation, which is the most frequently observed mutation in NS patients, provide a fundamental relationship between the NS-associated neurological manifestations and defective cortical development. Taken together, we propose that the intellectual and cognitive impairments of NS patients may originate from abnormalities in glutamatergic neurons during cortical development.

## 4. Materials and Methods

### 4.1. Maintenance of Human Induced Pluripotent Stem Cells (hiPSCs)

WT- and NS-iPSCs were previously generated from their respective dermal fibroblasts [24]. hiPSCs were maintained in hiPSC medium on mitomycin C (AG Scientific, San Diego, CA, USA)-treated mouse embryonic fibroblasts (feeder). The hiPSC medium consisted of DMEM/F-12 (Gibco, Thermo Fisher, Waltham, MA, USA) supplemented with 20% KnockOut™ serum replacement (Gibco), 1% penicillin-streptomycin (Gibco), 1% MEM non-essential amino acids solution, 0.1 mM β-mercaptoethanol (Sigma-Aldrich, Merck, Darmstadt, Germany), 1.2 mg/mL sodium carbohydrate (Sigma-Aldrich, St. Louis, MO, USA), and 10 ng/mL recombinant human bFGF (R&D Systems, Minneapolis, MN, USA). The hiPSCs were incubated at 37 °C in an atmosphere containing 5% CO_2_ with a daily medium change, and passaged every 6 days.

### 4.2. Genomic Correction of NS-iPSCs

sgRNA candidates and potential off-target sites for the *PTPN11* c.922A > G genomic locus were designed using CRISPR RGEN tools [58]. Sequence information for the sgRNA, ssODN, and primers is listed in Table S1. sgRNA, ssODN, and Cas9 vectors were transfected into NS-iPSCs, as previously described [59]. Briefly, 3 × 10^6^ NS-iPSCs were mixed with 7.5 μg of sgRNA vector, 7.5 μg of pCas9-GFP vector, and 15 μg of ssODN in 300 μL of Opti-MEM (Gibco). The cell mixture was transferred to three individual cuvettes and electroporated using a poring pulse of 125 V, 5 ms from a NEPA21 Super Electroporator (Nepagene, Shioyaki, Ichikawa, Japan). Transfected cells were cultured in mTeSR™1 medium (STEMCELL Technologies, Vancouver, BC, Canada) supplemented with 10 μM Y-27632 for 24 h, and then only GFP-positive cells were selected using BD FACSAria™ II Cell Sorter (BD Biosciences, Franklin Lakes, NJ, USA). Sorted cells were plated on the feeder-coated 6-well plates at the density of 500 cells per well and cultured in hiPSC medium supplemented with 10 μM Y-27632 until colonies formed. The correct colonies (NS-Cor-iPSCs) were identified by genomic sequencing (Macrogen, Seoul, Korea), and one NS-Cor-iPSC line exhibiting a normal pluripotent stem cell morphology was used in subsequent experiments.

### 4.3. Cortical Organoid Differentiation

hiPSCs were differentiated into cortical organoids as previously reported, but with minor modifications [60]. Briefly, hiPSC colonies were mechanically cut into four to nine clumps of approximately 0.5 mm × 0.5 mm in size using a syringe needle and treated with 10 mg/mL collagenase type IV (Gibco) for 4 min. Detached clumps were cultured in suspension on low-attachment dishes in EB medium supplemented with 10 μM Y-27632 (Cayman Chemical, Ann Arbor, MI, USA) for 1 day, after which the medium was changed daily to fresh EB medium without Y-27632 for 4 days. The EB medium was composed of hiPSC medium without bFGF, supplemented with 10 μM SB431542 (Cayman Chemical, Ann Arbor, MI, USA) and 10 μM dorsomorphine (AG Scientific, San Diego, CA, USA). The EB-derived spheres were further incubated in neural medium containing Neurobasal™-A medium (Gibco), 2% B-27™ supplement minus vitamin A (Gibco), 1% GlutaMAX™ supplement (Gibco), 20 ng/mL bFGF, and 20 ng/mL recombinant human EGF (Peprotech, Cranbury, NJ, USA) for 10 days with a daily medium change, and for 9 days with the medium changed every other day. From days 25 to 43 of culture, cortical organoids were incubated in neural medium supplemented with 20 ng/mL recombinant human BDNF (Peprotech) and 20 ng/mL recombinant human NT-3 (Peprotech) instead of EGF and FGF, with the medium changed every other day. For prolonged culture, cortical organoids were incubated in neural medium without any growth factors, with the medium changed every other day. The morphology of cortical organoids was imaged on an inverted microscope (Olympus, Tokyo, Japan), and their surface areas were analyzed with ImageJ software (NIMH, Bethesda, MD, USA)

### 4.4. Real-Time Quantitative PCR

Total RNAs were extracted from COs by using easy-BLUE™ Total RNA Extraction Kit (Intron Biotechnology, Seongnam, Korea). cDNA was synthesized from 1 μg of each RNA with the First Strand cDNA Synthesis Kit (Bioassay, Daejeon, Korea). The real-time quantitative PCR (qPCR) was performed on a CFX-Connect Real-Time System (Bio-Rad Laboratories, Hercules, CA, USA) through 40 cycles of 95 °C denaturation for 20 s, 60 °C annealing for 15 s, and 72 °C elongation for 15 s. The sequence of primers used in this study were GAPDH-forward: GAAGGTGAAGGTCGGAGTC, GAPDH-reverse: GAAGATGGTGATGGGATTTC, PTPN11-forward: GTGGAGGAGAACGGTTTGATTC, and PTPN11-reverse: CCAATGTTTCCACCATAGGATTC. The expression of *PTPN11* gene was normalized by the expression of *GAPDH*. Relative gene expression level of each sample was calculated using the formula 2^Ct(GAPDH)-Ct(PTPN11)^.

### 4.5. Western Blotting

COs were lysed in RIPA buffer (GenDEPOT, Katy, TX, USA) by homogenizing with VCX-750 ultrasonic processor (Sonics & Materials, Newtown, CT, USA). Concentration of each protein lysate was measured by using Pierce™ BCA Protein Assay Kit (Thermo Fisher, Waltham, MA, USA) and equalized in the concentration of 1 μg/μL. Protein lysates were denatured by boiling at 95 °C with an addition of 5X SDS-PAGE Loading Buffer (LPS solution, Daejeon, Korea). Protein samples (10 μL) were run on 10% SDS-polyacrylamide gel and then transferred to a nitrocellulose membrane (Merck). Membranes were blocked with 3% BSA solution at RT for 1 hr and then incubated in the blocking solution containing respective primary antibodies at 4 °C for overnight. The primary antibodies used were SHP2 (#3397; 1:500, Cell Signaling Technologies, Danvers, MA, USA), p-SHP2 (#3751; 1:500, Cell Signaling Technologies, Danvers, MA, USA), ERK (#9102; 1:1000, Cell Signaling Technologies, Danvers, MA, USA), and p-ERK (#9101; 1:1000, Cell Signaling Technologies, Danvers, MA, USA). The membranes were then incubated with HRP-conjugated anti-rabbit antibody (#7074; 1:1000, Cell Signaling Technologies, Danvers, MA, USA) and GAPDH-HRP (sc-47724; 1:1000, Santa Cruz Biotechnology, Dallas, TX, USA) at RT for 1 hr. Bands of respective proteins were detected on iBright™ CL750 Imaging System (Thermo Fisher, Waltham, MA, USA). The intensity of each band was analyzed using the ImageJ software.

### 4.6. Single-Cell RNA Sequencing (scRNA-seq) Library Preparation

Four individual cortical organoids of similar size were obtained from respective samples (WT, NS, and NS-Cor) at different developmental periods (weeks 8-, 16-, and 24) and subjected to scRNA-seq library preparation. Each cortical organoid was dissected into small pieces using a 29G-syringe needle and dissociated using the papain dissociation system (Worthington Biochemical Corporation, Lakewood, NJ, USA) according to the manufacturer’s protocol. Dissociated cells were suspended in 1 mL PBS solution supplemented with 0.04% BSA and 10 μM Y-27632, counted using an UNA-II automated cell counter (Logos Biosystems, Anyang, Korea), and diluted at a concentration of 1 × 10^6^ cells/mL. A scRNA-seq library was prepared by using Chromium single-cell 3’ reagent kits v3.1 chemistry (10× Genomics, Pleasanton, CA, USA). Generated scRNA-seq libraries were sequenced on the Novaseq 6000 sequencing system (Illumina, San Diego, CA, USA). The targeted read depth was 20,000 reads per cell for week-8 and week-16 samples, and 40,000 reads per cell for week-24 samples.

### 4.7. Analysis of scRNA-seq Data

Reads were aligned to the GRCh38 human reference genome and converted into count matrices using the Cell Ranger 6.1.2 pipeline. Count matrices were further processed by Seurat 4.0.6 [61]. Cells expressing less than 1000 or more than 5500 genes, less than 2000 or more than 20,000 unique molecular identifiers, or a mitochondrial transcript proportion higher than 20% were filtered out for quality control. *MALAT1* and mitochondrial gene expression was regressed out to avoid biases caused by variations in mitochondrial gene expression. Each dataset was log-normalized and linear transformed. The top 30 principal components (PCs) obtained by principal component analysis (PCA) with 2000 highly variable features were subjected to further analysis. Nearest neighbors were calculated, and the cells were initially clustered using the Louvain algorithm at a resolution of 0.5. DEGs in each cluster were identified using the Wilcoxon test. Clusters displaying apoptosis-associated DEGs were excluded from further analysis. Nine individual Seurat objects were integrated using the Harmony package [62] for batch-correction. The integrated dataset was clustered and visualized by UMAP dimensional reduction. Clusters were annotated according to their respective cell types using canonical cell type-specific marker genes. The top ten hallmarks for the GO:BP and KEGG gene sets were analyzed using the fGSEA package [63]. RNA velocity analysis was performed using the velocyto package [64]. A subset of cells lining the EN developmental pathway were analyzed using the Monocle 3 package (v.1.0.0) to construct single-cell trajectories [65]. 

### 4.8. Immunostaining

COs were fixed in 4% formaldehyde at 4 °C overnight, washed twice in PBS, and cryopreserved in 30% buffered sucrose solution (Sigma-Aldrich, St. Louis, MO, USA) at 4 °C for 72 h. Samples were then embedded in Tissue-Tek^®^ O.C.T. Compound (Sakura Finetek, Torrance, CA, USA) and stored at −80 °C until cryosection. Frozen COs were cut into 20 μm thick slices on a Leica CM1850 cryostat (Leica biosystems, Nussloch, Germany) and placed on Superfrost™ Plus Microscope Slides (Thermo Fisher, Waltham, MA, USA). For immunostaining, the samples were blocked in PBS supplemented with 3% BSA and 0.5% Triton X-100 at RT for 2 h, and then incubated with the primary antibodies diluted in blocking solution at 4 °C overnight. The primary antibodies used were PAX6 (1:100, DSHB, Iowa City IA), TBR2 (HPA028896; 1:200, Atlas Antibiodies, Bromma, Sweden), MAP2 (M1406; 1:1000, Sigma-Aldrich), SOX2 (#3579; 1:400, Cell Signaling Technologies, Danvers, MA, USA), SATB2 (ab51502; 1:500, Abcam, Cambridge, UK), CTIP2 (ab18465; 1:500, Abcam, Cambridge, UK), PSD95 (#3450; 1:200, Cell Signaling Technologies, Danvers, MA, USA); and Synaptophysin (ab8049; 1:100, Abcam, Cambridge, UK). After rinsing three times with PBST, the samples were incubated with Alexa 488- or 594- conjugated secondary antibody (1:1000, Abcam, Cambridge, UK) with DAPI at RT for 2 h. Immunostained images were then acquired on Zeiss LSM800 and LSM980 confocal microscopes (Carl Zeiss, Oberkochen, Germany). To visualize the cortical layer, merged z-stack images were rotated to align the layer boundary horizontally and cropped to a width of 100 μm. CTIP2^+^ or SATB2^+^ positive cells were counted using the “Cell counter” plugin in ImageJ software. Areas showing co-localization of the presynaptic marker synaptophysin and the postsynaptic marker PSD-95 were measured using Fiji software.

### 4.9. Multi-Electrode Array (MEA)

Eighteen COs (week 24) developed from WT-, NS-, and NS-Cor-iPSCs were subjected to MEA analysis. COs were attached to a PLO/laminin-coated CytoView MEA 48 plate (Axion Biosystems, Atlanta, GA, USA) at a density of one organoid per well, and cultured in neural medium for one week. Spontaneous spike recording was performed using Axion Maestro Pro (Axion Biosystems, Atlanta, GA, USA) for 15 min and repeated three times at an interval of 30 min. The recording was performed every other day for two weeks. Spontaneous spike recording data were analyzed using the Axis navigator program (Axion BioSystems, Atlanta, GA, USA). Raster plots for the representative MEA recording were visualized using the Neural metric tool (Axion BioSystems, Atlanta, GA, USA). To determine the number of spikes and bursts, electrodes with an electrophysiological signal frequency of less than five spikes per minute during the recording were removed. 

### 4.10. Statistics

Organoid differentiation experiments were repeated at least three times. The organoid surface area is presented as the means ± standard deviation (SD). The data obtained from all other experiments are presented as means ± standard error of the mean (SEM). Statistical significance was analyzed with the two-tailed Student *t*-test; * *p* < 0.05, ** *p* < 0.01, *** *p* < 0.001.

## Data Availability

The scRNA-seq data of WT-/NS-/NS-Cor-COs used in this study are deposited in the GEO database. These data are available under accession number GSE213798.

## Supplementary Materials

The following supporting information can be downloaded at: https://www.mdpi.com/article/10.3390/ijms232213861/s1.
