# Supplementary material for: Aberrant Cortical Layer Development of Brain Organoids Derived from Noonan Syndrome-iPSCs
Source: Int J Mol Sci. 2022 Nov 10;23(22):13861. doi: 10.3390/ijms232213861 (PMC9699065; doi:10.3390/ijms232213861)
Supplement: Supplementary file 1 [file ijms-23-13861-s001.zip › ijms-1959351-supplementary.pdf]

Supplementary Materials

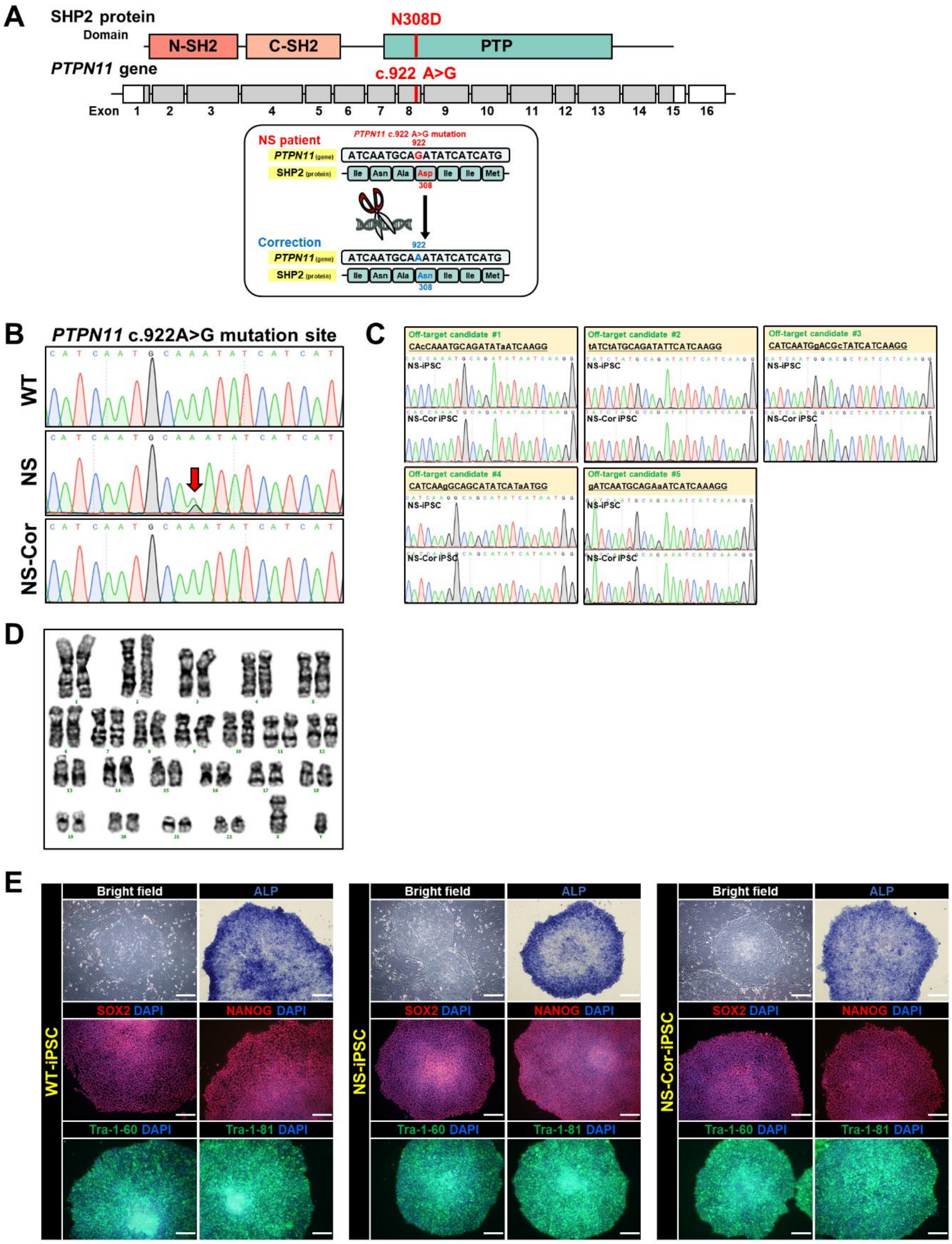

Figure S1. Genomic correction of NS-iPSCs using the CRISPR/Cas9 system. **(A)**

Schematic illustration of the genomic correction procedure **(B)** Successful *PTPN11* c.922G>A conversion in NS-Cor-iPSCs **(C)** Top 5 off-target site sequence integrity analysis **(D)** A normal karyotype was observed in NS-Cor-iPSCs **(E)** Normal morphology and expression of pluripotency markers in WT-/NS-/NS-Cor-iPSCs. Scale bars, 200 μm.

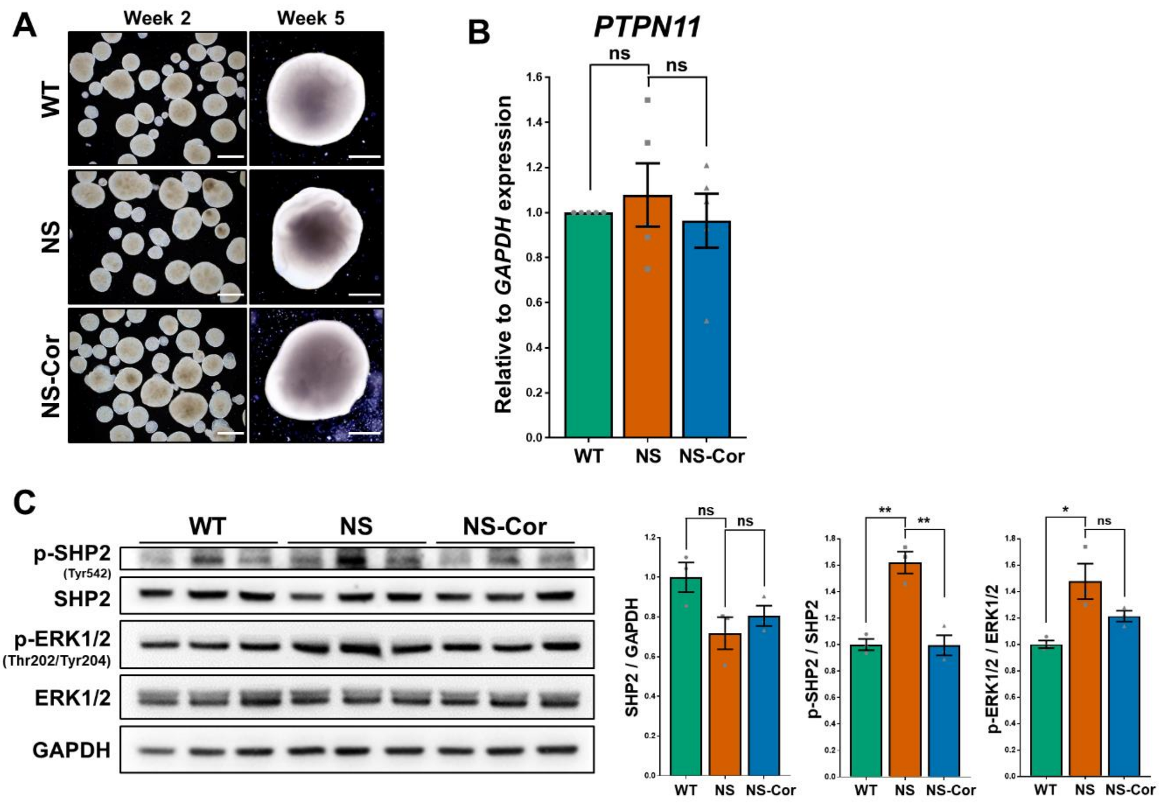

Figure S2. Upregulated MAPK pathway in NS-COs. **(A)** Normal morphology of WT-/NS-/NS-Cor-COs during early development. Scale bars, 500 μm. **(B)** Unaffected *PTPN11* mRNA level in NS-COs. COs for qPCR analysis were obtained from five individual experiments. **(C)** Increased SHP2 phosphorylation and MAPK pathway activation in NS-COs. COs for western blot analysis were obtained from three individual experiments. The relative ratio of protein expression was quantified with ImageJ. In G–H, data are presented as means ± SEM. P-values were determined using an unpaired Student's t-test. *P< 0.05; **P< 0.01.

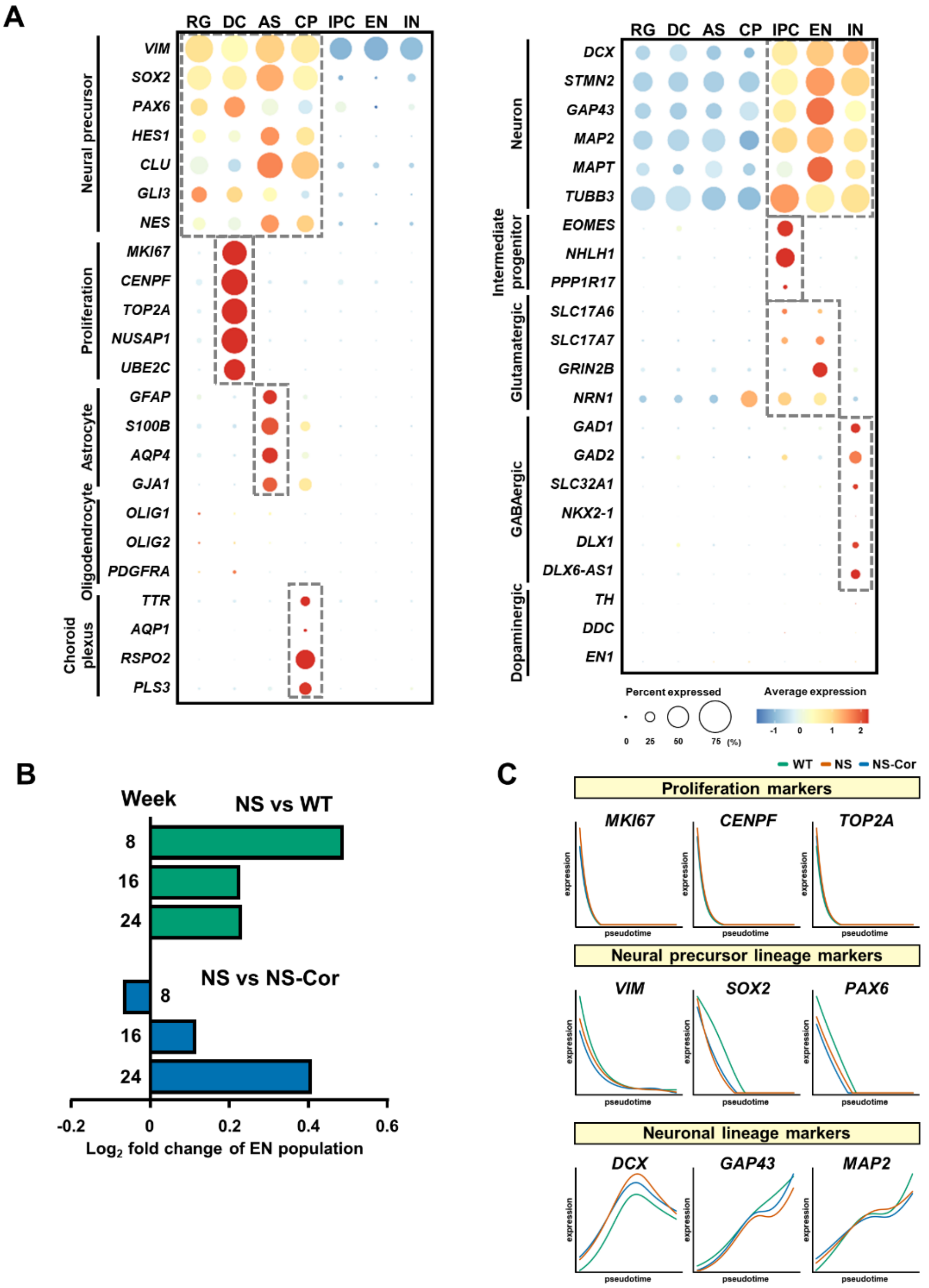

Figure S3. Increased EN population in NS-COs. (A) Expression of additional lineage- and cell type-specific marker genes in each cluster (B) Differences in the proportions of ENs in the respective CO-groups. The proportion of ENs in NS-COs at each time point is divided by the proportion of ENs in WT- and NS-Cor-COs, and its fold change is log-transformed. (C) Transcriptional expression of proliferation-, neural

precursor lineage-, and neuronal lineage-associated markers along the pseudotime.

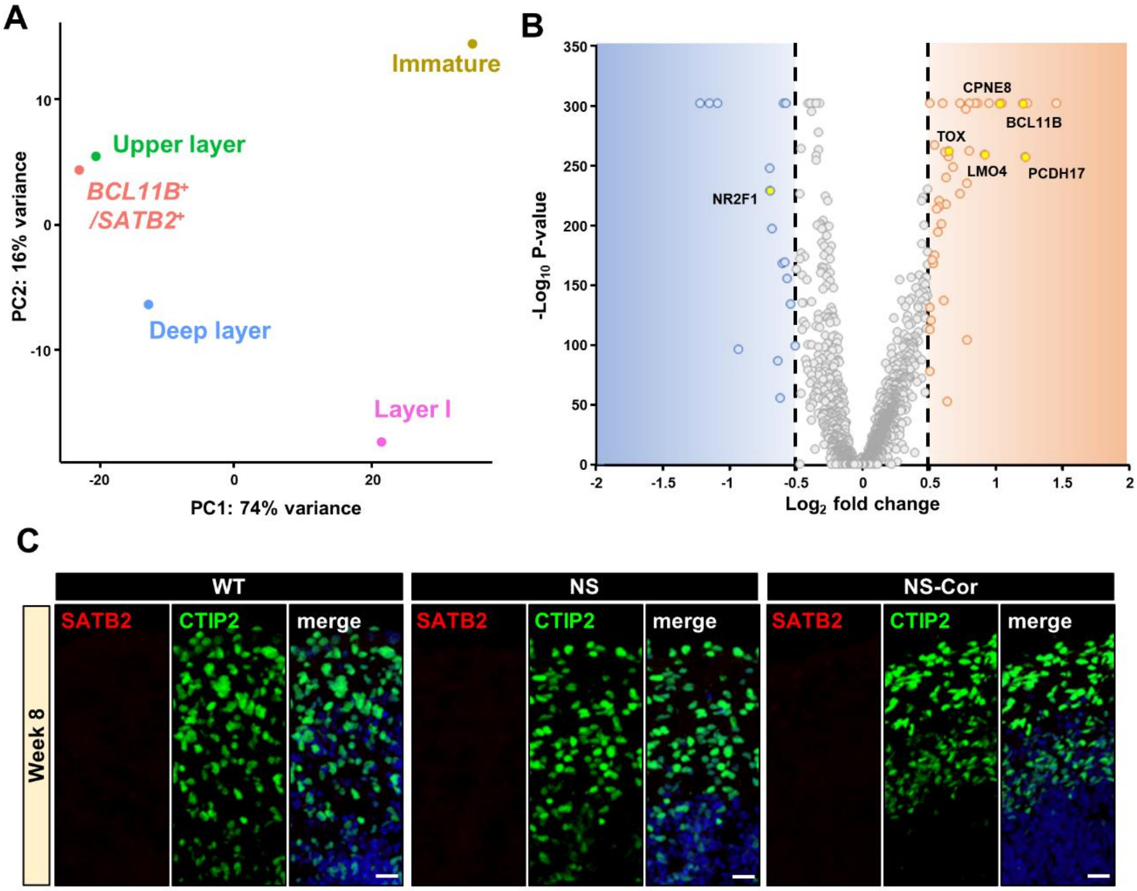

Figure S4. Similar transcriptional profiles between upper layer EN and *BCL11B*⁺/*SATB2*⁺ EN. (A) Principal component analysis of each pseudobulked-layer EN population. (B) Volcano plot showing up- and downregulated DEGs of *BCL11B*⁺/*SATB2*⁺ EN versus upper layer EN. (C) Undetected SATB2 expression in all week 8-COs. Six organoids per group was obtained from three independent experiments. Scale bars, 20 μm

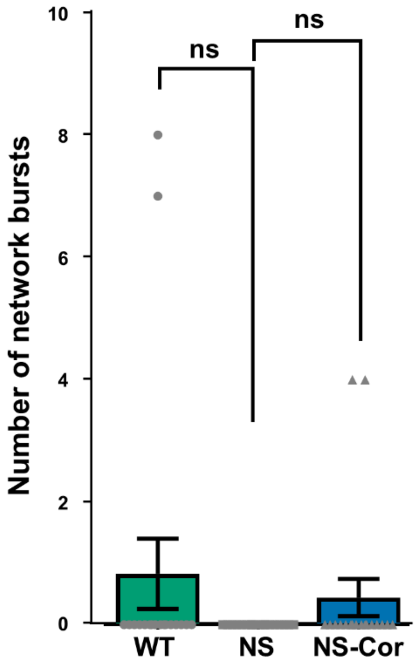

Figure S5. Number of network bursts in respective COs. The number of network bursts was counted in 18 MEA wells and acquired using Axis navigator software. Data are presented as means ± SEM. *P*-values were determined using an unpaired Student's *t*-test. ns, not significant.

Table S1. Sequence information for the sgRNA, ssODN, and primers used in genomic correction.

sgRNA	*CATCAATGCAGATATCATCATGG*	
ssODN	*ACCGTGGTCTCTTTTTCTTCTAGTTGATCATACCAGGGTTGTCCT ACACGATGGTGATCCCAATGAGCCTGTTTCAGATTACATCAATGC AAATATCATTATGCTAAGCTTTGCTTTTCACAGTGTT*	
PTPN11 mutation site primer	*F*	*TTTTCCTGAAGCAGTCCAG*
	R	*ATCCGCCAAAAGTCATTCAC*
PTPN11 off-target primer#1	*F*	*TGAACAACACACAAGGGCCT*
	R	*TGTGCATGTTGAACCAACCT*
PTPN11 off-target primer#2	*F*	*CAGAGGCAGAGGGGAAACAG*
	R	*CTGCTTCCCTCCTTACCGTG*
PTPN11 off-target primer#3	*F*	*ACCTTCCTGCCATGCATTCA*
	R	*CCAAAGAATAGGGAGGCGCA*
PTPN11 off-target primer#4	*F*	*TGAGGTGGAGGAAGAAGGGA*
	R	*GAGCAAGACTCCATCCCTGG*
PTPN11 off-target primer#5	*F*	*TCCAGGCTGTGACACACATC*
	R	*ATGGGCCTCACAACATCCAG*
